# Antimicrobial and Synergistic Effects of Syzygium cumini, Moringa oleifera, and Tinospora cordifolia Against Different Candida Infections

**DOI:** 10.7759/cureus.52857

**Published:** 2024-01-24

**Authors:** Adedayo O Adelakun, Ayoola Awosika, Uzochukwu Adabanya, Adekunle E Omole, Akinyode I Olopoda, Emmanuel T Bello

**Affiliations:** 1 Biological Sciences, Southeast Iowa Regional Medical Center, West Burlington, USA; 2 College of Medicine, University of Illinois, Chicago, USA; 3 Anatomical Sciences, Edward Via College of Osteopathic Medicine, Monroe, USA; 4 Cell Biology and Anatomy, Louisiana State University, Health Science Center, New Orleans, USA; 5 Biochemistry, Federal University of Technology Akure, Akure, NGA; 6 Science Laboratory Technology, New Land Polytechnic, Ilorin, NGA

**Keywords:** tinospora cordifolia, syzygium cumini, moringa oleifera, fluconazole, candida, multidrug resistance

## Abstract

Introduction

The burden of multiple drug resistance in human pathogens has necessitated the search for and development of antimicrobial agents with a wide range of structural classes and potentials to selectively act on the several mechanisms of actions exhibited by the pathogens. However, most synthetic antimicrobial agents have been linked with adverse side effects and high costs, furthering the need to explore more options. *Syzygium cumini*,* Moringa oleifera*, and *Tinospora cordifolia* are three medicinal plants used in traditional medicine systems for various infectious diseases. They contain various phytochemicals that exhibit antimicrobial activities against various bacteria, fungi, and parasites. The mechanisms of their antimicrobial action may involve the disruption of microbial cell walls and membranes, the inhibition of microbial enzyme and biofilm formation, the modulation of microbial gene expression and quorum sensing, and the induction of microbial cell death. Therefore, the present study evaluated the potentials of aqueous and ethanol extracts of *S. cumini*, *M. oleifera*, and *T. cordifolia *in managing infections as measured by their inhibitory effects on species.

Materials and method

*Syzygium cumini*, *M. oleifera*, and *T. cordifolia* were obtained and authenticated, and their aqueous and ethanol extracts were prepared. The antibacterial properties of the aqueous and ethanol extracts were examined. In addition to broth microdilution and biofilm development experiments, we also employed disk diffusion and agar-well diffusion techniques. The inocula of various species, including *krusei*, *parapsilosis*,* utilis*, *albicans*, and *glabrata*, were prepared for these assays. The synergistic effect of plant extracts with fluconazole was also evaluated.

Results

*Syzygium cumini*, *M. oleifera*, and *T. cordifolia *emerge as promising sources for the development of effective and sustainable antimicrobial interventions. Interestingly, the aqueous and ethanol extracts were effective against the selected species. Also, the synergistic combination of plant extracts with fluconazole was observed to triple the potency of the extracts. Furthermore, the potency of the plant extract as an antifungal and synergistic agent was ranked as *S. cumini* > *M. oleifera* > *T. cordifolia*. Conclusively, the plant extracts are effective in the management of opportunistic fungal infections.

## Introduction

Opportunistic fungal pathogens are major causes of infection in immunocompromised individuals such as surgical patients or critically ill patients [1]. Of these opportunistic fungal pathogens, the most common are *Candida* species, which are the primary sources of systemic and mucosal infection in humans [2]. Although this fungus occurs as a normal flora in animals and humans, it may become opportunistic and produce lethal and disabling infections [3]. *Candida* infection is characterized by high death rates and exorbitant healthcare costs for patients and governments [4]. The prevalent mortality rate as a result of *Candida* infection has been attributed to the ever-growing rate of invasive systemic infections as well as septicemia cases, particularly in immunocompromised individuals [5]. Interestingly, 90% of invasive infections have been linked to different species of opportunistic *Candida*, making it the fourth principal cause of nosocomial bloodstream infections [6].

Analytically, the last decade has recorded as high as 20% of *Candida* infections attributed to these non-*albicans* *Candida* species [7]. Of the non-*albicans* *Candida* species, *Candida glabrata* has the highest frequency of infection [8]. It is not only the least sensitive *Candida* species to antifungal drugs but also the most difficult to eliminate. Conversely, *Candida parapsilosis* is mostly considered the least virulent *Candida* species, and it is capable of frequently causing candidemia in health workers with poor hand hygiene [9]. *Candida krusei* is prevalent in individuals who are diagnosed with Down syndrome [10].

Resistance of *Candida* species to various antifungal drugs has resulted in immunosuppressed patients being subjected to extensive stays in intensive care units, surgery, and broad-spectrum antibiotic treatments. All of these have been observed to further elevate the possibility of disseminating candidiasis [4]. Regrettably, multidrug resistance (MDR) in human pathogenic microorganisms has developed due to the indiscriminate use of commercial antimicrobial drugs commonly used in treating infectious diseases. This situation has forced scientists to search for new antimicrobial substances from various sources as novel antimicrobial chemotherapeutic agents.

Having considered various possible approaches, exploring medicinal plants containing diverse bioactive compounds that have been employed for antifungal activity in folklore could be more productive in treating and managing candidiasis. This is because natural products from these medicinal plants have been explored and observed to exhibit unlimited opportunities in the treatment of candidiasis owing to their bioavailability of chemical diversity [11]. *Syzygium cumini*, *Moringa oleifera*, and *Tinospora cordifolia* are three medicinal plants rooted in traditional medicine that exhibit broad-spectrum antimicrobial activity against bacteria, fungi, and viruses [11]. The bioactive compounds within each plant, including ellagic acid, quercetin, and tannins in *S. cumini*, isothiocyanates and chlorogenic acid in *M. oleifera*, and alkaloids and glycosides in *T. cordifolia*, contribute to their antimicrobial efficacy. Abdelgadir et al. reported that *S. cumini* extracts possess in vitro antibacterial activities and could be used not only in traditional medicine. He suggested that further research work should be carried out on this plant to determine the toxicity as well as the optimum dose. The result should then be compared with standard antibiotics [12].

The mechanisms of their antimicrobial action may involve the disruption of microbial cell walls and membranes, the inhibition of microbial enzyme and biofilm formation, the modulation of microbial gene expression and quorum sensing, and the induction of microbial cell death [11]. Beyond their direct antimicrobial effects, these botanicals hold promise for therapeutic applications in infectious diseases, wound healing, and immune support. Further research is warranted to elucidate specific molecular mechanisms, optimize formulations, and validate their clinical efficacy. Therefore, this study seeks to comparatively evaluate edible and safe-for-consumption plants, such as *S. cumini*, *M. oleifera*, and *T. cordifolia*, in the management of *Candida* infections, as measured by their inhibitory effects on *Candida* species.

## Materials and methods

Sample collection and preparation

Fresh leaves of *S. cumini*, *M. oleifera*, and *T. cordifolia* were obtained from local farmers in Akure South Local Government, Ondo State, Nigeria. The leaves were authenticated with voucher number (0347) obtained at the Centre for Research and Development (CERAD), Federal University of Technology, Akure, Nigeria. The leaves were then sorted out of debris, washed, air-dried, and pulverized into fine textured powder using Warring Commercial Heavy-Duty Blender (Model 37BL18; 24ØCB6) to ensure better surface contact with extraction solvents. The pulverized samples were then prepared according to modified methods [13]. Typically, 20 g of the powdered samples were weighed with a weighing balance (Sartorius BP61S) and dissolved in 100 mL of distilled water (for aqueous extract) and 70% ethanol (for ethanol extract). This was macerated for 24 hours and filtered into a conical flask with Whatman's No. 1 filter paper to get a clear filtrate. Subsequently, each extract was shaken on a vortex mixer to facilitate dilution. The filtrate was kept in the refrigerator at 4°C for further use. This was repeated every week to get a fresh filtrate. The concentrated extracts were further diluted by mixing with appropriate phosphate buffer saline (PBS) and dimethyl sulfoxide (DMSO). Stock solutions of aqueous/ethanolic extracts using the appropriate aseptic technique were further diluted by dissolving 100 μL of the aqueous/ethanolic extracts in 200 μL of PBS/5% of DMSO to get a 1:2 dilution and 100 μL of the aqueous/ethanolic extracts in 400 μL of PBS/5% of DMSO to get a 1:4 dilution, respectively.

Standard drug preparation

Fluconazole, used as a positive control, was dissolved in 5% DMSO to give a stock concentration of 10 mg/mL. Fluconazole, a well-established antifungal drug, is often used as a positive control in antimicrobial assays at varying concentrations than those used for the plant extracts being studied. This is a result of certain reasons that include its known efficacy, which negates the need for higher concentrations such as those used for experimental extracts. It serves as a benchmark for making comparisons and needs to be distinctly identifiable in its effects. Fluconazole's potential toxicity at higher concentrations also necessitates its use at lower concentrations. Moreover, differences in solubility and modes of action between fluconazole and plant extracts may require disparate concentrations to effectively demonstrate their respective antimicrobial effects.

Inoculum preparation

Reference strains of Candida (*C. krusei* American Type Culture Collection [ATCC] 14243, *C. parapsilosis* ATCC 22019, *C. utilis* and *C. albicans* ATCC 76615, and *C. glabrata* ATCC 66032) were obtained from Coventry University Super Laboratory. These were then grown in Sabouraud (SAB) dextrose agar for 18 hours at 37°C as previously described by Vasanthi et al. [14]. The handling and use of the Candida were approved by the CERAD Ethical Committee of the Federal University of Technology with the ethical number (FUTA/ETH/2020/016). The *C. krusei*, *C. parapsilosis*, *C. utilis*, *C. albicans*, and *C. glabrata* isolates were maintained in the SAB dextrose (Thermo Scientific™ Oxoid) agar all throughout the study duration. Each of the Candida species (*C. krusei*, *C. parapsilosis*, *C. utilis*, *C. albicans*, and *C. glabrata*) from their stock cultures was streaked on agar plates and then incubated at 37°C for 24 hours. Two yeast colonies from each of the agar plates were emulsified in sterile SAB broth (Oxoid, CM0147) as inocula [15]. It was incubated at 37°C for 24 hours, and the resulting solution was vortexed for 10 seconds. Afterward, the turbidity was adjusted with a spectrophotometer (Biochrom Ltd, Cambridge, England) to adjust it to 0.5 McFarland standard at 530 nm wavelength. Exactly 0.5 McFarland gives an equivalent approximate density of yeast cells 1.5 × 10^8^ CFU mL^− 1^ [16].

Evaluation of the antifungal activities of plant extracts against *Candida* species

Disk Diffusion Method

The evaluation of *S. cumini*, *M. oleifera*, and *T. cordifolia* activities was carried out on *C. krusei*, *C. parapsilosis*, *C. utilis*, *C. albicans*, and *C. glabrata* using the disk diffusion method as reported by Okla et al. [15]. SAB dextrose agar (SDA) was poured into the Petri dishes, and each of the Petri dishes was then loaded with 100 µL of the inocula (*C. krusei*, *C. parapsilosis*, *C. utilis*, *C. albicans*, and *C. glabrata*). Typically, these 6 mm diameter sterile filter paper disks were obtained and inserted into Petri dishes containing 20 µL of plant extracts. The different concentrations (100%, 50%, and 25%) of *S. cumini*, *M. oleifera*, and *T. cordifolia* were loaded via a 0.22 mm Millipore filter over the filter paper disks. Distilled water and ethanol only (without plant extracts) served as the negative controls, while fluconazole (10 mg/mL) was the positive control. DMSO was used as the solvent for the ethanol extracts, and PBS was used as the solvent for the aqueous extracts. All the Petri dishes used in the disk diffusion method were maintained in a 5°C environment for 2 hours during the diffusion of plant extract and then incubated for 24 hours at 37°C anaerobically. The incubation results on each Petri dish were documented after the zone of inhibition had been measured. Each test was carried out in triplicate, and the average values were recorded.

Agar-Well Diffusion Method

As employed by Hirsch et al. [17] with a slight modification, inoculum susceptibility tests were carried out on fresh agar plate cultures provided using a sterile swab stick to spread the yeast culture throughout the plates and allowed to dry. The agar plate was divided into sections and labeled accordingly. Five wells were made using a sterile cork borer (6 mm in diameter) into agar plates containing inocula and were filled with 100 µL of plant extract of 100%, 50%, and 25% concentration separately; 50 µL of three concentrations (100%, 50%, and 25%) of *S. cumini*, *M. oleifera*, and *T. cordifolia* were added to the wells. It was allowed to stay for 1 hour at room temperature before incubating. Fluconazole (10 mg/mL) served as a positive control in another well. With the lids uppermost, the plates were incubated for 24 hours at 37°C. Inhibition zones of the extracts against the fungi were measured in millimeters (mm) and interpreted by comparing them against the Clinical and Laboratory Standards Institute (CLSI). The values were presented means of three independent experiments.

Broth Microdilution Assay

Microdilution was performed as recommended by the CLSI [18]. Therefore, the minimum inhibitory concentration (MIC) of *S. cumini*, *M. oleifera*, and *T. cordifolia* plant extracts that exhibited strong antifungal activity against selected species of *Candida* (*C. krusei*, *C. parapsilosis*, *C. utilis*, *C. albicans*, and *C. glabrata*) was investigated. Various concentrations of the stock, 0.78 to 200 mg/mL, were assayed against the test fungi. For the broth microdilution test, 100 µL of each fungal suspension in the suitable growth medium was added to the wells of a sterile 96-well microtiter plate already containing 100 µL of two-fold serially diluted plant extract in Mueller Hinton agar. Column 1 was a control to monitor sterility, while columns 2-11 contained 100 µL each of inoculated broth and plant extracts. A multichannel pipette was then used to transfer and mix biosurfactants from columns 2-11, resulting in 100 µL per well, and 100 µL taken from the last well was discarded. The final volume in each well was 100 µL. Control wells were prepared with culture medium, fresh broth suspension, and plant extracts only in amounts corresponding to the highest quantity present. The plates were incubated at 37°C for 48 hours. All measurements of MIC values were repeated in triplicate. The MIC was determined by checking for turbidity using a spectrophotometer [19].

Biofilm Formation Using the Resazurin Dye Method

The inhibitory potentials of the undiluted concentrations of the plant extracts against the biofilm formation of Candida species were evaluated using the resazurin reduction method as described by Gómez-Casanova et al. [20]; 0.015g of resazurin powder (R7017-1G Sigma-Aldrich, Inc, St. Louis, MO, USA) was dissolved in 100 mL of diluted PBS X10 to get a final concentration of 0.015%. It was sterilized by filtration (Whatman’s filter paper) and stored at 4°C until use. The preparation procedures were performed in the dark, and the resazurin solution was then kept in a foil-wrapped bottle to prevent exposure to light due to its sensitivity to light. The inoculum was prepared per the CLSI recommendation. Under aseptic conditions, with a 96-well plate, 100 µL of SAB broth and plant extract were dispensed in each well of column 1 as a control to monitor sterility, while columns 2-11 contained 100 µL each of inoculated broth and plant extracts. A multichannel pipette was then used to transfer and mix biosurfactants from columns 2 to 11, resulting in 100 µL per well, and 100 µL taken from the last well was discarded. Column 12 was used as a positive control with inoculum only and incubated for 48 hours at 37°C. According to the protocol used by Elshikh et al. [21], 0.015% resazurin was added to all wells (30 µL per well) and further incubated for 3 to 4 hours to observe color change. The reduction of resazurin, therefore, correlates with the number of live cells. The transference of electrons from NADPH to resazurin is expected to reduce the blue resazurin to a pink, fluorescent counterpart, resorufin. On completion of the incubation, columns were read spectrophotometrically at 595 nm.

Synergistic effect of plant extracts with antibiotics

Synergistic antifungal activity was measured using a good diffusion method according to the National Committee for Clinical Laboratory Standards [22, 23]. Under aseptic conditions, Petri dishes containing approximately 20-25 mL of SDA medium were inoculated using a cotton swab. The plants, separately powdered, were extracted by adding 24 hours of old culture of the fungal strains. Wells (6 mm diameter) were punched in the agar and filled with 30 µL of plant extracts or antibiotics, but in case of synergism effect, 30 µL of each extract and antibiotic was added into the wells. Replicates of each plate were done. The plates were incubated at 37°C for 24 hours. The antifungal activity was assessed by measuring the inhibition zone diameter (mm) around the well. The average of three replicates for each extract, antibiotic, and combination was calculated. The synergism effect was considered when combinations were exhibited with fluconazole (10 mg/mL).

Statistical analysis

The results from the present study were collected and graphed for statistical analysis using Microsoft Excel and Statistical Package for Social Science (SPSS) Software Version 26 (IBM Corp., Armonk, NY, USA). The effects of the different extractants at the different selected dilution concentrations (100%, 50%, and 25%) were evaluated against the *Candida* species. Liquid dilution, as well as biofilm formation, was evaluated via univariate one-way analysis of variance (ANOVA), and the significance differences were obtained at p < 0.05. The inhibition zones were calculated as means ± SD of three replicates. The whole procedure was repeated thrice to ascertain the validity of the obtained results.

## Results

Agar-well diffusion

The aqueous extract of *T. cordifolia* did not show activity against any of the five targeted fungal species for all concentrations used (Table 1). However, the mean zone of inhibition measured (in mm) of 100% concentration ethanol extract of *T. cordifolia* against *C. albicans*, *C. glabrata*, and *C. utilis* were 15.33 ± 0.57, 16.67 ± 0.57, and 14.67 ± 0.57, respectively; 100% aqueous extract of *M. oleifera* exhibited a mean zone of inhibition (mm) of 16.00 ± 0.00, 17.00 ± 0.00, and 18.00 ± 0.00, respectively, against fungal species mentioned above. Similarly, the mean zones of inhibitions, measured in mm, were 28.67 ± 0.57, 25.33 ± 0.57, 26.67 ± 1.15, 30.33 ± 0.57, and 25.67 ± 1.15 against *C. albicans*, *C. glabrata*, *C. utilis*, *C. parapsilosis*, and *C. krusei*, respectively, for *S. cumini* (100% water extract), while the corresponding values for the 100% ethanol extract of *S. cumini* were 27.00 ± 0.00, 28.33 ± 0.57, 31.00 ± 1.00, 34.00 ± 1.00, and 29.00 ± 1.00 (Table 1). Likewise, the zones of inhibition (mm) for 50% water extract concentration of *S. cumini* against *C. albicans*, *C. glabrata*, *C. utilis*, *C. parapsilosis*, and *C. krusei* were measured to be 21.00 ± 1.00, 22.33 ± 1.15, 20.67 ± 0.57, 19.00 ± 1.00, and 18.00 ± 0.00, respectively. However, the same concentration for the ethanol extract of *S. cumini* against *C. albicans*, *C. glabrata*, *C. utilis*, *C. parapsilosis*, and *C. krusei* were measured to be 25.67 ± 0.57, 25.00 ± 1.00, 26.00 ± 0.00, 26.00 ± 1.00, and 26.67 ± 1.15, respectively (Table 1). *C. parapsilosis* and *C. krusei* were seen to be susceptible to 50% and 25% ethanol extract concentrations of *M. oleifera*, with the mean zones of inhibition measuring (mm) 26.33 ± 0.57, 21.00 ± 0.00 and 21.00 ± 0.00, 12.33 ± 1.52, respectively. It was found that the antifungal activities increased with concentration of extracts. Syzygium cumini showed antifungal activity against all five fungal species unlike *T. cordifolia* and *M. oleifera*. The negative controls (DMSO and PBS) did not inhibit any of the microorganisms tested. However, positive control (10 mg/ mL of fluconazole) exhibited a mean zone of inhibition (mm) of 24.00 ± 0.57, 40.00 ± 1.00, and 25.00 ± 0.57 against *C. glabrata*, *C. parapsilosis*, and *C. krusei*, respectively, as shown in Table 1.

**Table 1:** Mean zones of inhibition of plant extracts against Candida species NI, no inhibition; Conc, concentration.

Candida spp.	Plant extracts (mm)							Positive control (10 mg/mL)
	Conc (%)	Tinospora cordifolia		Moringa oleifera		Syzygium cumini		
		Water	Ethanol	Water	Ethanol	Water	Ethanol	
C. albicans	100	NI	15.33 ± 0.57	16.00 ± 0.00	NI	28.67 ± 0.00	27.00 ± 0.00	NI
	50	NI	0.00 ± 0.00	NI	NI	21.00 ± 1.00	25.67 ± 0.57	
	25	NI	NI	NI	NI	18.67 ± 0.57	25.00 ± 1.00	
C. glabrata	100	NI	16.67 ± 0.57	17.00 ± 0.00	NI	25.33 ± 0.57	28.33 ± 0.57	24.00 ± 0.57
	50	NI	NI	NI	NI	22.33 ± 1.15	25.00 ± 1.00	
	25	NI	NI	NI	NI	16.00 ± 0.00	26.00 ± 2.00	
C. utilis	100	NI	14.67 ± 0.57	18.00 ± 0.00	NI	26.67 ± 0.57	31.00 ± 1.00	NI
	50	NI	NI	NI	NI	20.67 ± 0.57	26.00 ± 0.00	
	25	NI	NI	NI	NI	15.00 ± 0.00	24.00 ± 0.00	
C. parapsilosis	100	NI	NI	NI	31.67 ± 1.52	30.33 ± 0.57	34.00 ± 1.00	40.00 ± 1.00
	50	NI	NI	NI	26.33 ± 0.57	19.00 ± 1.00	26.00 ± 1.00	
	25	NI	NI	NI	21.00 ± 0.00	17.00 ± 0.00	24.67 ± 1.52	
C. krusei	100	NI	NI	NI	22.00 ± 1.72	25.67 ± 1.15	29.00 ± 1.00	25.00 ± 0.57
	50	NI	NI	NI	21.00 ± 0.00	18.00 ± 0.00	26.67 ± 1.15	
	25	NI	NI	NI	12.33 ± 1.52	16.00 ± 0.00	23.00 ± 2.00	
Negative control		NI	NI	NI	NI	NI	NI	

**Figure 1:**
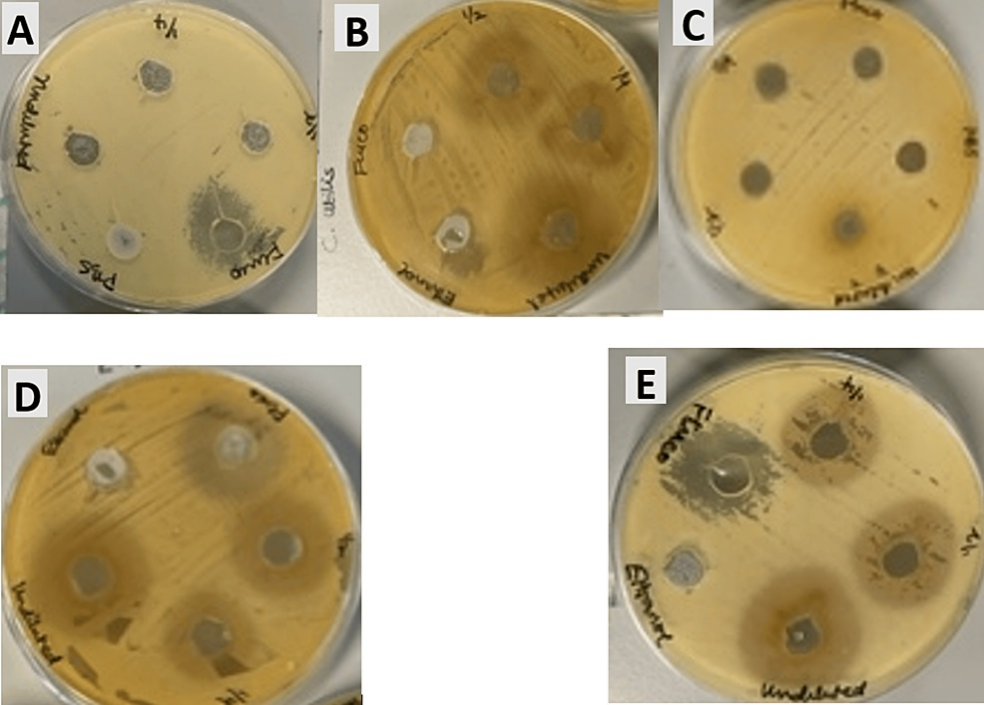
Plate 1. Zone of inhibition at different concentrations of different plant extracts against Candida species by well diffusion method. (A) *Candida glabrata*. (B) *Candida albicans*. (C) *Candida utilis*. (D) *Candida parapsilosis*. (E) *Candida krusei*. As labelled on the plates: Undiluted, 100% concentration; ½, 50%; ¼, 25%; Fluco, fluconazole; PBS/DMSO, negative control. PBS, phosphate buffer saline; DMSO, dimethyl sulfoxide.

The difference in activity of the plant extracts was statistically non-significant (p > 0.05). The activity of *S. cumini* differed significantly (p < 0.05) from the rest of the extracts. Overall, the ethanol extracts exhibited the highest mean zone diameter (mm) of 34.00 ± 1.00, as shown in Figure 1. Plant extracts of *S. cumini* had the best activity recorded against all fungal strains used.

Disc diffusion assay

The aqueous and ethanol extracts of *S. cumini* proved to be effective against all *Candida* species tested, while these *Candida* species did not show any effect toward *T. cordifolia* and *M. oleifera* even with the highest concentrations. The results showed maximum activity against *C. parapsilosis* at 100% concentrations of water and ethanol by *S. cumini* with the mean zone of inhibition (mm) measuring 15.00 ± 1.00 and 16.33 ± 0.57, respectively, which was lower than the control value (Table 2). However, the inhibition zones of *S. cumini*, when treated with 50% and 25% concentrations on the same isolate, were 10.33 ± 0.57 and 9.33 ± 0.57 for water and 13.00 ± 1.15 and 10.33 ± 0.57 for ethanol, respectively. Nevertheless, *T. cordifolia* and *M. oleifera* showed no activity against *C. parapsilosis*. Only *S. cumini* exhibited an inhibitory effect on *C. albicans* with a mean zone of inhibition (mm) measuring 11.00 ± 1.00, 9.00 ± 0.00, and 9.00 ± 1.00 for water extracts, while water extracts show 15.00 ± 1.00, 12.00 ± 1.00, and 8.00 ± 1.00 for 100%, 50%, and 25% concentrations, respectively. Not all the isolates used in the study were inhibited. However, the antifungal activity varied with species. *Candida krusei* and *C. parapsilosis* were the most inhibited species, and *C. albicans* was the least inhibited species. Aqueous extracts were a bit less active than the ethanolic extracts in all concentrations. The zone of inhibition recorded at 100% concentration was higher than that of 50% and 25% concentration for all the extracts. As the amount of the extract increased, the inhibitory effect also increased. Fluconazole (10 mg/mL), used as the positive control, produced a zone of inhibition of 10.00 ± 0.57, 23.00 ± 1.00, and 15.00 ± 0.00 only on *C. glabrata*, *C. parapsilosis*, and *C. krusei*, respectively. However, there was no significant activity against other *Candida* species; 5% of DMSO and PBS negative control was used and did not have any inhibitory effect on any of the five isolates of *Candida* species tested, demonstrating a total lack of antimicrobial activity. The results showed that both aqueous and ethanol extracts of *S. cumini* were found to be more effective against all the fungi tested (p < 0.05) compared to other extracts.

**Table 2:** Mean zones of inhibition of plant extracts against Candida species Note: The mean value (n = 3) is presented as mean ± SD. The one-way ANOVA was used to determine the average zones of inhibition, which were reported as mean ± SD. Key: The negative control is 5% DMSO for ethanol and PBS for water extracts; the positive control used was fluconazole. NI, no inhibition; Conc, concentration

Candida spp.	Plant extracts (mm)							Positive control (10 mg/mL)
	Conc (%)	Tinospora cordifolia		Moringa oleifera		Syzygium cumini		
		Water	Ethanol	Water	ethanol	Water	Ethanol	
C. albicans	100	NI	NI	NI	NI	11.00 ± 1.00	15.00 ± 0.00	NI
	50	NI	NI	NI	NI	9.00 ± 0.00	12.00 ± 1.00	
	25	NI	NI	NI	NI	9.00 ± 1.00	8.00 ± 0.00	
C. glabrata	100	NI	NI	NI	NI	13.00 ± 0.00	13.33 ± 1.15	10.00 ± 0.57
	50	NI	NI	NI	NI	8.67 ± 0.57	10.00 ± 1.00	
	25	NI	NI	NI	NI	7.00 ± 1.00	8.00 ± 0.00	
C. utilis	100	NI	NI	NI	NI	14.00 ± 1.00	15.33 ± 0.57	NI
	50	NI	NI	NI	NI	7.67 ± 0.57	12.00 ± 0.00	
	25	NI	NI	NI	NI	7.00 ± 1.00	8.00 ± 0.00	
C. parapsilosis	100	NI	NI	NI	NI	15.00 ± 1.00	16.33 ± 0.57	23.00 ± 1.00
	50	NI	NI	NI	NI	10.33 ± 0.57	13.00 ± 0.00	
	25	NI	NI	NI	NI	9.33 ± 0.57	10.33 ± 0.57	
C. krusei	100	NI	NI	NI	NI	12.67 ± 0.57	15.00 ± 1.00	15.00 ± 0.00
	50	NI	NI	NI	NI	9.00 ± 0.00	12.33 ± 1.15	
	25	NI	NI	NI	NI	8.00 ± 0.00	10.00 ± 1.00	
Negative control		NI	NI	NI	NI	NI	NI	

**Figure 2:**
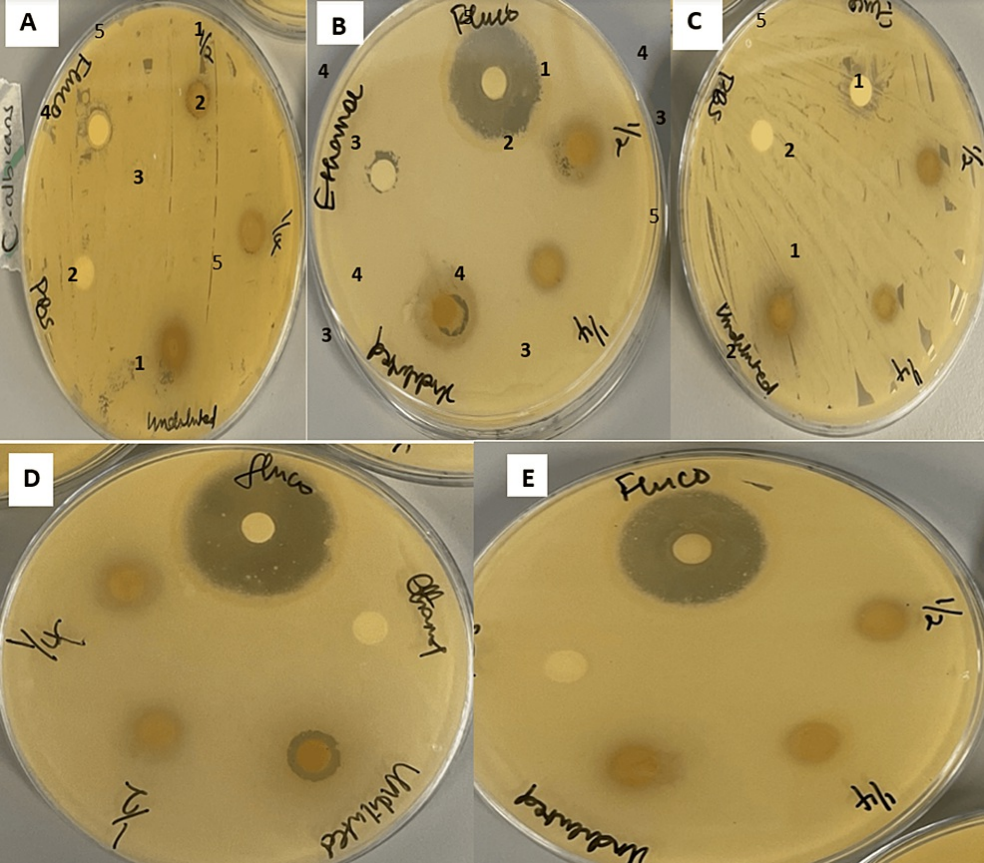
Plate 2. Antimicrobial activity of ethanol extract of different concentrations of Syzygium cumini on Candida species by disk diffusion method. (A) *Candida albicans*. (B) *Candida glabrata*. (C) *Candida utilis*. (D) *Candida parapsilosis*. (E) *Candida krusei*. 1, 50%; 2, 25%; 3, 100%; 4, growth control (70% ethanol); 5, fluconazole (negative control, 10 mg/mL).

In summary, the analysis shows a significant difference in the MIC among the groups being studied, as shown in Figure 2. Given the p-value of 0.000, the results are highly significant at the 0.05 level. The use of fluconazole as a positive control provides a reference point for the comparison.

Broth microdilution assay

The effectiveness of the plant extracts in the *Candida* strains was confirmed by measuring the MICs. MICs of plant extracts for the organisms were determined using the broth dilution method. Results indicated different MIC levels based on the fungal strains being tested. MICs of aqueous and ethanol extracts ranged from 0 to 100 mg/mL. MICs of aqueous and ethanol extracts ranged from 0 to 100 mg/mL. MIC values ranged from 0.15 to 100 mg/mL for *C. glabrata*, 0 to 50 mg/mL for *C. utilis*, and 0 to 100 mg/mL for *C. albicans*, *C. parapsilosis*, and *C. krusei*.

In ethanolic extracts of the three plants, MIC ranged from 0.39 to 100 mg/mL, and the range for aqueous extracts was the same. *Syzygium cumini* recorded the lowest MICs for all tested fungal strains with aqueous and ethanolic extracts. For ethanolic extracts of *S. cumini*, the MICs were 3.13, 6.25, 0.39, 0.78, and 1.56 mg/mL in *C. albicans*, *C. glabrata*, *C. utilis*, *C. parapsilosis*, and *C. krusei*, respectively, while the MICs for its aqueous extracts were 1.56, 0.78, 0.78, 0.39, and 0.78 mg/mL in *C. albicans*, *C. glabrata*, *C. utilis*, *C. parapsilosis*, and *C. krusei*, respectively (Table 3). *Tinospora cordifolia* showed the weakest MICs among all tested extracts. Its aqueous extract had MIC values of 100, 50, 0, 100, and 0 mg/mL in *C. albicans*, *C. glabrata*, *C. utilis*, *C. parapsilosis*, and *C. krusei*, respectively, while the ethanol extract had values of 25, 25, 50, 0, and 100 mg/mL in *C. albicans*, *C. glabrata*, *C. utilis*, *C. parapsilosis*, and *C. krusei*, respectively. *Moringa oleifera* also showed a weak MIC among all tested extracts. Its aqueous extract had MIC values of 0, 25, 50, 50, and 0 mg/mL in *C. albicans*, *C. glabrata*, *C. utilis*, *C. parapsilosis*, and *C. krusei*, respectively, while the ethanol extract had values of 50, 100, 50, 12.50, and 12.50 mg/mL in *C. albicans*, *C. glabrata*, *C. utilis*, *C. parapsilosis*, and *C. krusei*, respectively (Table 3). The p-value was <0.001. There is a statistically significant difference between the MIC means and plant species tested.

**Table 3:** MIC values of aqueous and ethanol extracts (mg/mL) of T. cordifolia, M. oleifera, and S. cumini against Candida species Note: There was a two-fold serial dilution with 100 mg/mL at initial concentration. Fluconazole was used as a positive control. 0, no inhibition activity

	Plant MIC (mg/mL)						Fluconazole (10 mg/mL)
	Tinospora cordifolia		Moringa oleifera		Syzygium cumini		
	Water	Ethanol	Water	Ethanol	Water	Ethanol	
C. albicans	100.00	25.00	0.00	50.00	1.56	3.13	10.00
C. glabrata	50.00	25.00	25.00	100.00	0.78	6.25	0.15
C. utilis	0.00	50.00	50.00	50.00	0.78	0.39	5.00
C. Parapsilosis	100.00	0.00	50.00	12.50	0.39	0.78	0.039
C. krusei	0.00	100.00	0.00	12.50	0.78	1.56	0.78

The presented tables offer insights into the antifungal activities of three plants - *T. cordifolia*, *M. oleifera*, and *S. cumini* - against several *Candida* species. The MIC values highlight the effectiveness of the plant extracts in inhibiting the growth of *Candida* species. A lower MIC value indicates a stronger antifungal effect. Ethanol extracts generally seem more effective than water extracts, especially in the case of *T. cordifolia* against *C. albicans* and *C. parapsilosis*. Interestingly, the water extract of *M. oleifera *shows no inhibitory activity against *C. albicans* and *C. krusei*, whereas its ethanol counterpart demonstrates significant activity.

Fluconazole, a standard antifungal drug, is included as a positive control, and its varying MIC values against different *Candida* species offer a benchmark for comparison. For instance, *S. cumini's* ethanol extract displays notable potency against *C. glabrata *with an MIC of 0.15 mg/mL, which is notably lower than that of fluconazole for the same species.

**Figure 3:**
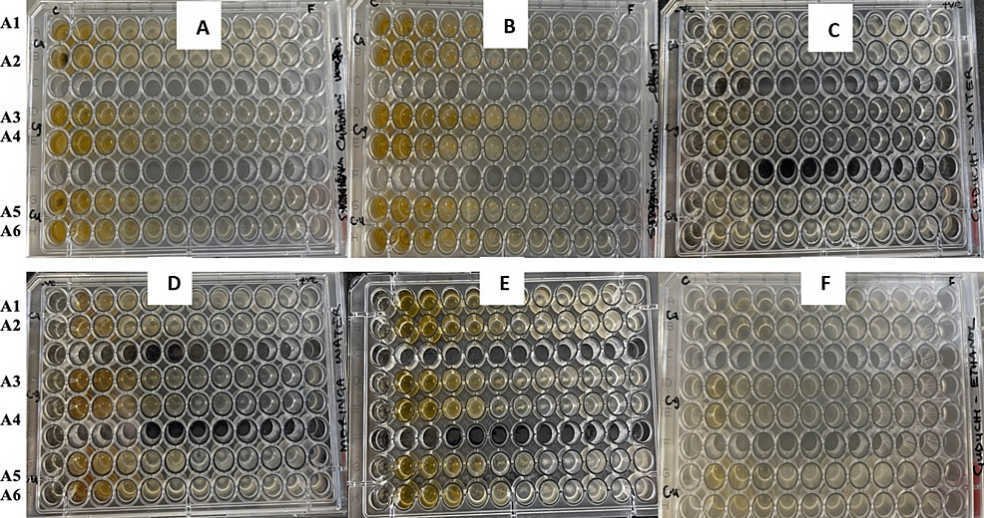
Plate 3. Determination of minimum inhibitory concentration of plant extracts on Candida species by microdilution method. (A) *Syzygium cumini* water. (B) *Syzygium cumini* ethanol. (C) *Tinospora cordifolia* water. (D) *Moringa oleifera* water. (E) *Moringa oleifera* ethanol. (F) *Tinospora cordifolia* ethanol extract. C = growth control; C+ = positive control; 2 = 100; 3 = 50; 4 = 25; 5 = 12.5; 6 = 6.25; 7 = 3.125; 8 = 1.56; 9 = 0.78; 10 = 0.39; 11 = 0.195 (mg/mL)

The subsequent ANOVA analysis underscores a statistically significant difference in MIC values among the groups studied, as shown in Figure 3. This supports the notion that the effectiveness of these plant extracts - and their method of extraction - varies when it comes to inhibiting different *Candida* species. The statistical significance emphasizes the potential therapeutic promise and warrants further investigation into the active constituents responsible for the antifungal activities of these plants.

Resazurin-based turbidometric assay

In the resazurin-based turbidometric (RBTB) assay, all sterility control wells for all tested fungi remained blue in color after an overnight incubation followed by 4 hours of incubation with resazurin. In contrast, all wells in the growth test columns (containing growth medium and fungi) of all tested fungi changed color from blue to pink or from blue to pale pink. However, there was no consistency in the growth. There was an inconsistent increase and decrease in all samples. The growth percentage was calculated using univariate ANOVA for each *Candida* species based on the extracts.

Synergistic effect

**Figure 4:**
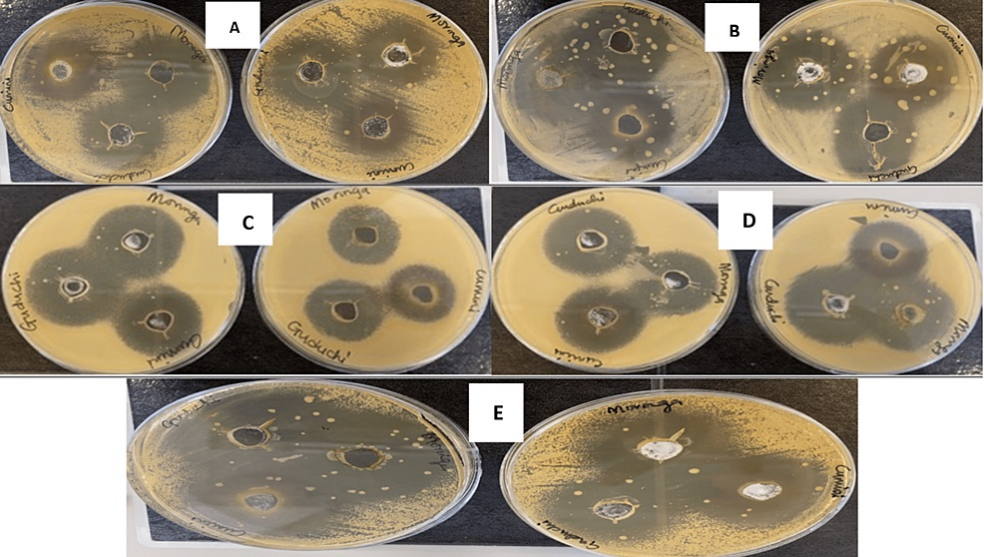
Plate 4. Image showing synergy efficacy of plant extracts mixed with fluconazole against Candida species. (A) *Candida albicans.* (B) *Candida utilis.* (C) *Candida krusei.* (D) *Candida parapsilosis.* (E) *Candida glabrata.* Negative control = 70% ethanol; positive control = fluconazole 10 mg/mL.

The synergistic effect of plants and fluconazole was evaluated using an agar-well diffusion assay. The results of the synergistic activity of extracts with antibiotics were determined by the diameters of inhibition zones, which are presented in Figure 4. All combinations of fluconazole with the plant extracts (water and ethanol) showed a synergistic effect on all fungal strains used. Of all plants tested, *C. glabrata* showed the greatest susceptibility with a mean zone of inhibition (mm) of 36.00 ± 0.00 and 45.00 ± 0.00 for *T. cordifolia* and fluconazole, respectively, 39.00 ± 0.00 and 43.00 ± 0.00 for *M. oleifera* and fluconazole, respectively, and 38.00 ± 0.00 and 42.00 ± 0.00 for S. cumini and fluconazole for water and ethanol extracts, respectively. In water extraction, the combinations of *T. cordifolia* and fluconazole had the highest combination against *C. albicans*, with a mean zone of inhibition (mm) of 33.33 ± 0.57. However, in ethanol extraction against *C. albicans*, *M. oleifera* and fluconazole combination showed more synergy effect with a mean zone of inhibition (mm) of 37.33 ± 0.57. The ethanolic extracts showed a more synergistic effect with fluconazole against fungal strains than the aqueous extracts, although a lesser effect was shown in the ethanolic extracts against *C. krusei* for the three plants. Among the five yeasts used in this research, the most resistant was *C. utilis*. Conversely, *C. parapsilosis* and *C. krusei* were the most sensitive strains to the tested extracts, as shown in Table 4. There was no significant difference in the zones of inhibition (p < 0.05 ). However, all combinations of extracts with fluconazole were found to be fungicidal against the five tested fungi compared to extracts alone and fluconazole alone, which had lesser zones of inhibition. This shows that the extracts had the ability to increase the action of fluconazole in these strains through synergism. Moreover, there is no significant difference in the mean zone of inhibition of the plants (Table 4).

**Table 4:** Mean zone of inhibition of plant extracts mixed with fluconazole against Candida species The mean values are expressed as mean ± SD (n = 3). Inhibition zones were measured in mm. Zones of inhibition include 6 mm for disk diameter.

Combinations		Fungal strain mean zone of inhibition				
		C. albicans	C. glabrata	C. utilis	C. parapsilosis	C. krusei
*T. cordifolia *and* Fluco*	Water	33.33 ± 0.57	36.33 ± 0.57	31.00 ± 0.00	30.67 ± 0.57	31.00 ± 0.00
	Ethanol	32.33 ± 0.57	45.00 ± 0.00	37.67 ± 0.57	38.67 ± 0.57	30.00 ± 0.00
*M. oleifera *and* Fluco*	Water	31.00 ± 0.00	39.33 ± 0.57	37.67 ± 0.57	30.00 ± 0.00	34.67 ± 0.57
	Ethanol	37.33 ± 0.57	43.33 ± 0.57	40.00 ± 0.00	31.00 ± 0.00	28.33 ± 0.57
*S. cumini *and* Fluco*	Water	30.00 ± 0.00	38.00 ± 0.00	32.33 ± 0.57	31.33 ± 0.57	31.33 ± 0.57
	Ethanol	29.33 ± 0.57	42.00 ± 0.00	32.33 ± 0.57	32.00 ± 0.57	27.00 ± 0.00

## Discussion

The ability of medicinal plants to act as antifungal agents is attributed to their phytochemical constituents, such as saponins, alkaloids, cyanogenetic glycosides, steroids, terpenoids, and flavonoids [15]. Alkaloids are therapeutically useful due to their antispasmodic, bacterial, and analgesic effects [13]. Flavonoid is a hydroxylated phenolic substance that is commonly synthesized by plants in response to microbial infection and, therefore, has been evaluated to elucidate potent antimicrobial activities against a wide range of microorganisms. Its therapeutic activity has been linked to its ability to form complexes with soluble and extracellular proteins as well as bacterial cell walls [23]. Saponins are established antimicrobial agents as they can mediate the leakage of proteins and certain enzymes from invading pathogenic cells [24]. Besides, steroids possess antibacterial attributes as there is a relationship between membrane lipids and their sensitivity to steroidal compounds, which establishes the proposed mechanism of steroids’ antibacterial action via their association with membrane lipids to cause leakages from liposomes [25]. Interestingly, some bioactive phytochemicals are bioavailable in *S. cumini*, *M. oleifera*, and *T. cordifolia* plants [25-27].

The agar-well diffusion method has been widely employed to determine the antifungal activity of plant extracts. The aqueous extract of *T. cordifolia* did not show any activity against all five target fungal species for all concentrations used. Inferentially, the aqueous extracted phytochemical constituents of *T. cordifolia* lack sufficient or appropriate bioactive antifungal constituents that can be evaluated using the agar-well diffusion method [28]. Meanwhile, the aqueous extracts of *M. oleifera* had mean inhibition zones only at the highest percentage concentration (100%), which indicates that the plant’s aqueous extracts might possess limited bioactive antifungal constituents at lower concentrations. Also, the ethanolic extracts of *T. cordifolia* and *M. oleifera* could only inhibit some strains of the *Candida* species. However, *S. cumini* significantly inhibited all the *Candida* species. This trend of the result may indicate that despite the effectiveness of polar solvent extracts in antimicrobial activities, only *S. cumini* significantly showed a mean zone of inhibition across all investigated *Candida* strains.

Additionally, a significant reduction in fungal growth in terms of the zone of inhibition around the disk during the disk diffusion assay was observed. Typically, only the aqueous and ethanol extracts of *S. cumini* showed concentration-dependent effectiveness against all *Candida* species tested. This observation agrees with earlier reports that documented the antibacterial activities of *S. cumini* and implicated its high bioactive phytochemical components for its antimicrobial activities [25]. However, the chemical properties of extraction solvents play decisive roles in demonstrating plants’ antifungal properties, as the presence of the phytochemicals extracted by the solvents may relate to the diverse antifungal activities against the *Candida* strains [15]. *Syzygium cumini* ethanolic extracts were more effective against *Candida* strains than aqueous ones, suggesting that ethanol may extract more antifungal ingredients. However, *T. cordifolia* and *M. oleifera* extracts showed no effect, implying *Candida* resistance or insufficient bioactive compounds in these plants.

During the RBTB assay, all sterility control wells for all tested bacteria remained blue in color after overnight incubation, followed by 4 hours of incubation with resazurin. This suggests that the antifungal effects of the plant extracts on the *Candida* strains may not be appropriately evaluated using the RBTB assay. This further clarifies why the RBTB assay was unable to evaluate the antifungal effects of the plant extracts on the *Candida* species.

In addition, all combinations of fluconazole with the plant extracts (water and ethanol) showed a synergistic effect on all *Candida* species. The combination with fluconazole led to around three times the inhibitory effect of the plant extracts against the *Candida* species. This result substantiates the theory that the combination of antifungal agents against *Candida* species is more effective [29]. *Candida* glabrata showed the greatest synergistic activity, followed by a combination with *T. cordifolia*. Also, ethanolic extracts showed a more synergistic effect with fluconazole against fungal strains than aqueous extracts. This correlates with a previous study that implicated the high hydrophobicity ability of the ethanolic extracts in increased membrane fluidizing effects that disrupt short-range interactions and facilitate membrane leakage as the cause of the increased synergistic effects [23]. The different resistance patterns of the *Candida* species also validate previous studies that established that each strain of the *Candida* species. employs a different resistance mechanism [29].

The great potency of *S. cumini* extract, particularly its ethanolic extract, can be attributed to several factors that collectively contribute to its superior antifungal activity compared to *T. cordifolia* and *M. oleifera* extracts. This is in tandem with a study conducted by Khan et al. [30]. These factors include the concentration of bioactive compounds, such as phenolic compounds, flavonoids, tannins, and essential oils, in higher concentrations than the other extracts. Another factor is the choice of solvent used in the extraction of the bioactive compounds. Ethanol is often more effective in extracting a wide range of phytochemicals, including those with antifungal properties. For this reason, the ethanol extract of *S. cumini* may contain a higher concentration of bioactive compounds compared to those of *T. cordifolia* and *M. oleifera*. Selective antifungal activity can also be a factor responsible for variations shown in the efficacy of *S. cumini* compared with other plant extracts. Different plant extracts may exhibit varying degrees of specificity against different fungal species [22]. In the case of *S. cumini*, its extract shows remarkable efficacy against *C. glabrata*, as evidenced by the notably low MIC values. This selectivity may be attributed to specific bioactive compounds within the extract that interact more strongly with the cellular structures or mechanisms unique to *C. glabrata*.

This study had a few limitations despite providing valuable insight into the in vitro clinical relevance of the plant extracts and their potential therapeutic applications. It does not address the effects, as well as safety, on human subjects; hence, generalizability cannot be assumed in the human population. Therefore, further research should extend beyond in vitro assessment. Such investigations should include preclinical and clinical trials to validate and establish the safety of these plant extracts for use as therapeutic agents by humans. This multifaceted approach is essential to building a strong basis for the possible use of the extracts as medicinal agents.

## Conclusions

*Syzygium cumini, M. oleifera*, and *T. cordifolia* emerge as promising sources for the development of effective and sustainable antimicrobial interventions. Notably, *S. cumini* stands out as a particularly robust candidate, demonstrating superior antifungal properties against all tested *Candida* species as compared to the other plant extracts. The effectiveness of both aqueous and ethanolic extracts of these plants against the selected *Candida* strains suggests a great inhibitory potential possessed by the plant extracts. Considering factors such as production cost, bioavailability, and minimal side effects even at higher dosages, plant extracts emerge as compelling alternatives to expensive synthetic drugs, which are notorious for their adverse effects. Also, *S. cumini* ranked the most active antifungal and synergistic agent, followed by *M. oleifera*, an indication of their potential in the management of opportunistic fungal infections. However, studies that encompass in vivo assays that would further establish the antifungal potentials of the aqueous and ethanolic extracts are recommended. More human clinical trials and safety evaluations are also needed to further validate their efficacy and establish their optimal dosage and mode of administration.
